# Trends in biomaterials in Japan

**DOI:** 10.1080/14686996.2021.1969097

**Published:** 2021-09-15

**Authors:** Jun Nakanishi, Mitsuhiro Ebara

**Affiliations:** a Research Center for Functional Materials, National Institute for Materials Science, Tsukuba, Japan; b Department of Nanoscience and Nanoengineering, Waseda University, Tokyo, Japan; c Department of Graduate School of Pure and Applied Sciences, University of Tsukuba, Tsukuba, Japan

The Japanese Society for Biomaterials (JSB) [1] was established on 4 December 1978 by the President of JSB, Prof. Takeo Yokobori (Tohoku University), together with many distinguished professors in the fields of materials science, medicine, and dentistry. Since then, perspectives of biomaterials research and development have changed markedly over the half-century period. During this period, JSB has not only played a key role as the center for interdisciplinary studies on biomaterials science and its applied research in domestic society but also participated in overseas scientific meetings on biomaterials as a member of the International Union of Societies for Biomaterials Science and Engineering (IUSBSE). JSB has engaged in activities that cover a wide range of biomaterials science, from base materials to medical devices, through industry-academia-government collaboration. In particular, JSB holds academic lectures, grants scientific awards, publishes journals, and develops guidelines for medical devices in relation to clinical trials.

Due to the outbreak of the global pandemic of COVID-19 in 2020, conventional in-person interactions among researchers through meetings and workshops have been seriously hampered. Nevertheless, the research and development of cutting-edge biomaterials and technologies in Japan are kept ongoing rather productively, even under the social-distancing policy. In this Focus Issue, we have invited some actively working biomaterials researchers from Japan to introduce their most recent studies, demonstrating our indomitable energy in this world pandemic crisis. We hope that this opportune Focus Issue will contribute to the further development of biomedical engineering, which helps save millions of lives every day.
